# Transcriptome reprogramming during severe dehydration contributes to physiological and metabolic changes in the resurrection plant *Haberlea rhodopensis*

**DOI:** 10.1186/s12870-018-1566-0

**Published:** 2018-12-13

**Authors:** Jie Liu, Daniela Moyankova, Chih-Ta Lin, Petko Mladenov, Run-Ze Sun, Dimitar Djilianov, Xin Deng

**Affiliations:** 1 Key Laboratory of Plant Resources, Institute of Botany, Chinese Academy of Sciences, Beijing, 100093 China; 2 Abiotic Stress Group, Agrobioinstitute, Agricultural Academy, 1164 Sofia, Bulgaria; 3 Facility Horticulture Laboratory of Universities in Shandong, Weifang University of Science and Technology, Shouguang, 262700 China

**Keywords:** Desiccation tolerance, *Haberlea rhodopensis*, Hormone signaling pathway, Primary and secondary metabolism, Resurrection plant, Transcriptome

## Abstract

**Background:**

Water shortage is a major factor that harms agriculture and ecosystems worldwide. Plants display various levels of tolerance to water deficit, but only resurrection plants can survive full desiccation of their vegetative tissues. *Haberlea rhodopensis*, an endemic plant of the Balkans, is one of the few resurrection plants found in Europe. We performed transcriptomic analyses of this species under slight, severe and full dehydration and recovery to investigate the dynamics of gene expression and associate them with existing physiological and metabolomics data.

**Results:**

De novo assembly yielded a total of 142,479 unigenes with an average sequence length of 1034 nt. Among them, 18,110 unigenes were differentially expressed. Hierarchical clustering of all differentially expressed genes resulted in seven clusters of dynamic expression patterns. The most significant expression changes, involving more than 15,000 genes, started at severe dehydration (~ 20% relative water content) and were partially maintained at full desiccation (< 10% relative water content). More than a hundred pathways were enriched and functionally organized in a GO/pathway network at the severe dehydration stage. Transcriptomic changes in key pathways were analyzed and discussed in relation to metabolic processes, signal transduction, quality control of protein and DNA repair in this plant during dehydration and rehydration.

**Conclusion:**

Reprograming of the transcriptome occurs during severe dehydration, resulting in a profound alteration of metabolism toward alternative energy supply, hormone signal transduction, and prevention of DNA/protein damage under very low cellular water content, underlying the observed physiological and metabolic responses and the resurrection behavior of *H. rhodopensis*.

**Electronic supplementary material:**

The online version of this article (10.1186/s12870-018-1566-0) contains supplementary material, which is available to authorized users.

## Background

Environmental stress, especially drought, place limitations on plant distribution and crop production worldwide. Intensive breeding efforts, based on crosses with wild drought-tolerant relatives as potential sources of useful traits, are underway. At the same time, interest in a small group called resurrection plants is increasing, as these plants can withstand long periods of almost full desiccation and recover their vegetative system activities quickly and fully upon rehydration. This makes them useful models to study desiccation tolerance and potential sources of genes, related to these important phenomena [1–4]. Further efforts are needed to elucidate the mechanisms of desiccation tolerance in resurrection plants as a sustained dynamic process.

The Balkan endemic *Haberlea rhodopensis*, one of the few species of resurrection plants, native to Europe, has been the subject of intensive genetic studies in recent years [5–7]. Transcriptomic analysis of *H. rhodopensis* gene expression under conditions of normal watering, dehydration (50% relative water content; RWC), full desiccation (5% RWC) and rehydration has been performed previously [5]. We have shown that the dynamics of desiccation tolerance can be characterized by numerous changes in various processes at additional important time points during drying: the stages of slight (~ 65–75%) and severe (20–25% RWC) dehydration [8]. For example, at the onset of drying (at 75% RWC), an approximately 25-fold increase in jasmonic acid (JA) [9] occurs, coinciding with the start of the decrease in photosynthetic performance [10]. On the other hand, oxygen evolution in leaves of *H. rhodopensis* did not decrease until 20% RWC [8]. Our studies revealed that the levels of numerous compounds changed dramatically during desiccation, particularly before or around the severe dehydration stage (25% RWC), including total phenols, sugars, total glutathione, malondialdehyde, sucrose/fructose ratio, abscisic acid (ABA), salicylic acid (SA) and starch levels [9, 11, 12]. Dramatic changes in photosynthetic performance and energy supply, such as ATP and glycolytic intermediates, were also observed at this stage of dehydration [9–12]. This time point appears to be of crucial importance, as non-resurrection plants related to *H. rhodopensis* tolerated the loss of almost two-thirds of their water content, whereas further drying to 20% RWC was irreversible [11].

In an attempt to dissect the dynamic transcriptional regulation events preceding the dramatic physiological and metabolic changes that occur during desiccation in *H. rhodopensis*, we performed de novo transcriptome sequencing at new time points during drying from the fully hydrated to desiccated state: slight and severe dehydration stages.

## Results

### Transcriptome sequencing and assembly

Transcriptomic analysis of gene expression in plants that were regularly watered (fresh control, F), slightly dehydrated to 75% RWC (D75), severely dehydrated to 20% RWC (D20), fully dehydrated to < 10% RWC (DT) and fully rehydrated after DT (R) was performed using Illumina next-generation sequencing technology. De novo assembly yielded a total of 142,479 unigenes, with an average sequence length of 1034 nt and an N50 value of 1664 nt (Additional file 1: Table S1). Approximately 75% of all unigenes were novel to *H. rhodopensis*. The sequence length distribution of the unigenes is indicated in Additional file 2: Fig. S1a. The numbers of unigenes annotated using several databases, NCBI non-redundant protein sequences, NCBI nucleotide collection, Swiss-Prot, Kyoto Encyclopedia of Genes and Genomes (KEGG), the Clusters of Orthologous Group (COG) and Gene Ontology (GO), ranged from 91,753 to 51,046. Among the matching sequences, 61% showed the closest match to sequences of *Sesamum indicum*, a species belonging to the family Pedaliaceae, order Scrophulariales (Additional file 2: Fig. S1b) [13]. There were 46,922 transcripts with no sequence similarity to known genes (orphan genes).

### Analysis of differentially expressed genes (DEGs)

Differential gene expression analysis was performed to monitor transcriptomic variations in the plant under five dehydration treatments. From the 10 libraries, 41,279,554 to 55,613,670 clean sequence reads were obtained after trimming (Additional file 3: Table S2), which were sufficient for quantitative analysis of gene expression. Based on the fragments per kilobase of transcript per million mapped reads (FPKM) of the unigenes in each sample, 18,110 unigenes were identified as DEGs, of which approximately 40% (6877) were novel.

**Fig. 1:**
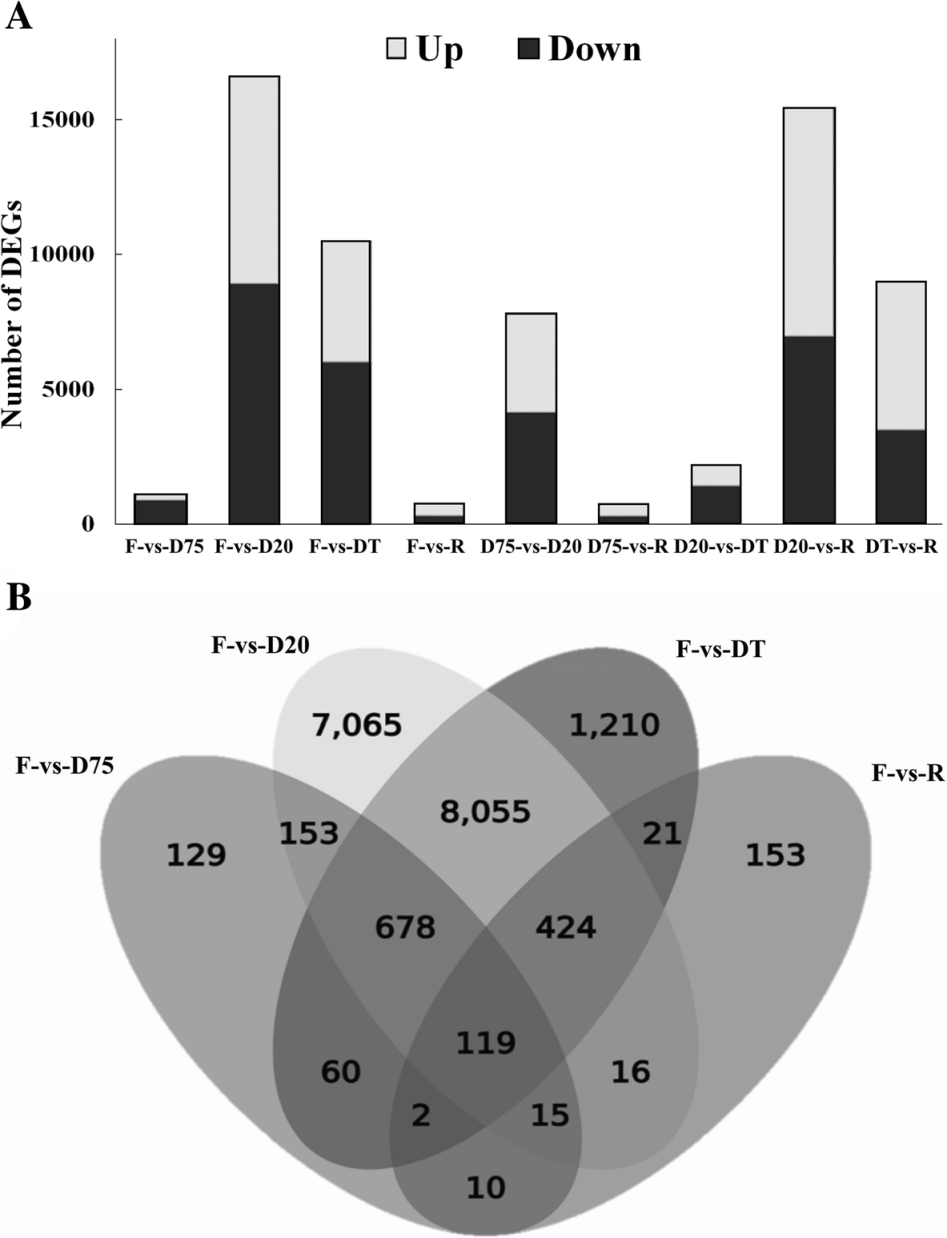
Differentially expressed genes (DEGs) in *Haberlea rhodopensis* in response to dehydration and rehydration. **a** The number of genes differentially expressed between two treatments according to a fold expression cutoff of 2 and an FDR ≤ 0.001. **b **Venn diagram illustrating the relationships among DEGs across the different treatments. F, Fresh control; D75, slightly dehydrated to 75% relative water content (RWC); D20, severely dehydrated to 20% RWC; DT, fully dehydrated to < 10% RWC; R, fully rehydrated after DT

Higher numbers of DEGs were observed in the comparisons of D20 with F (7,675 up and 8894 down), D75 (3.712 up and 4.120 down) or R (8,496 up and 6958 down) and in the comparisons of DT with F (4,527 up and 5998 down) or R (5,517 up and 3477 down). The lowest number of DEGs was found in the comparison of F with R (514 up and 290 down) (Fig. 1a). Among these DEGs, the highest levels of transcripts encoding LEA (late embryogenesis abundant) proteins, early light-inducible proteins, galactinol synthases, beta-amylases, sucrose synthases, senescence-associated proteins and raffinose synthases were seen at D20, whereas catalases were induced after slight dehydration (Additional file 4: Table S3).

A Venn diagram of the DEGs illustrates their occurrence in each dehydration treatment (Fig. 1b). The largest number of unique genes was found in the D20 (7065), followed by DT (1210), R (129) and D75 (153) treatments, whereas only 119 genes were present in all dehydration treatments. More than 80% of novel DEGs were either unique to D20 or shared by D20 and DT (Additional file 2: Fig. S1c). Based on their FPKM values, a random selection of 20 genes encoding enzymes, transcription factors and regulators involved in metabolism and stress responses were subjected to quantitative real-time PCR (qPCR) analysis to validate the DEGs identified by RNA-seq. The expression levels of these genes were significantly correlated with those determined by qPCR (*r* = 0.99, *p* < 0.01) (Additional file 5: Fig. S2a, Additional file 6: Table S4).

### Principal component analysis of transcript profiling of dehydrated and rehydrated plants

**Fig. 2:**
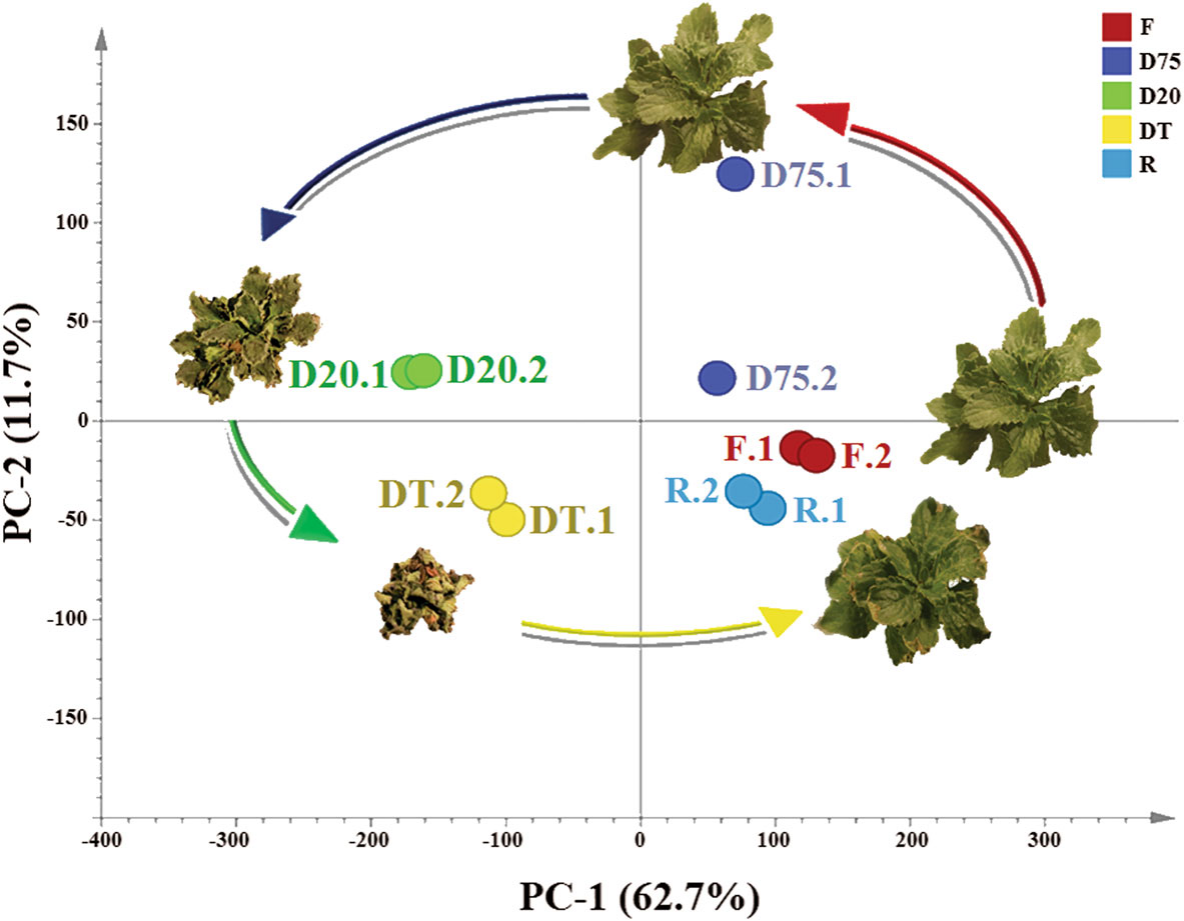
Principal component analysis (PCA) of transcriptomic sequencing data generated from the leaves of hydrated, desiccated and rehydrated *Haberlea rhodopensis* plants. Principal component (PC) 1 accounted for 62.7% of the variation, whereas PC2 accounted for 11.7% of the variation in the dataset. Plant samples are represented as different colored circles with sample abbreviations and photos. The clustering of dehydrated samples formed a resurrection cycle, indicated by the arrows. Sample abbreviations are the same as in Fig. 1

Principal component analysis was performed to capture the overall variance among samples. Samples in each treatment generated a cluster pattern according to water status (Fig. 2). The F, D75 and R samples were separated from the D20 and DT samples in principle component 1 (62.7%), whereas D75 was separated from F and R, and D20 from DT, in principle component 2 (11.7%).

### Clustering of DEGs by expression profiles and their correlations with physiological changes during dehydration

**Fig. 3:**
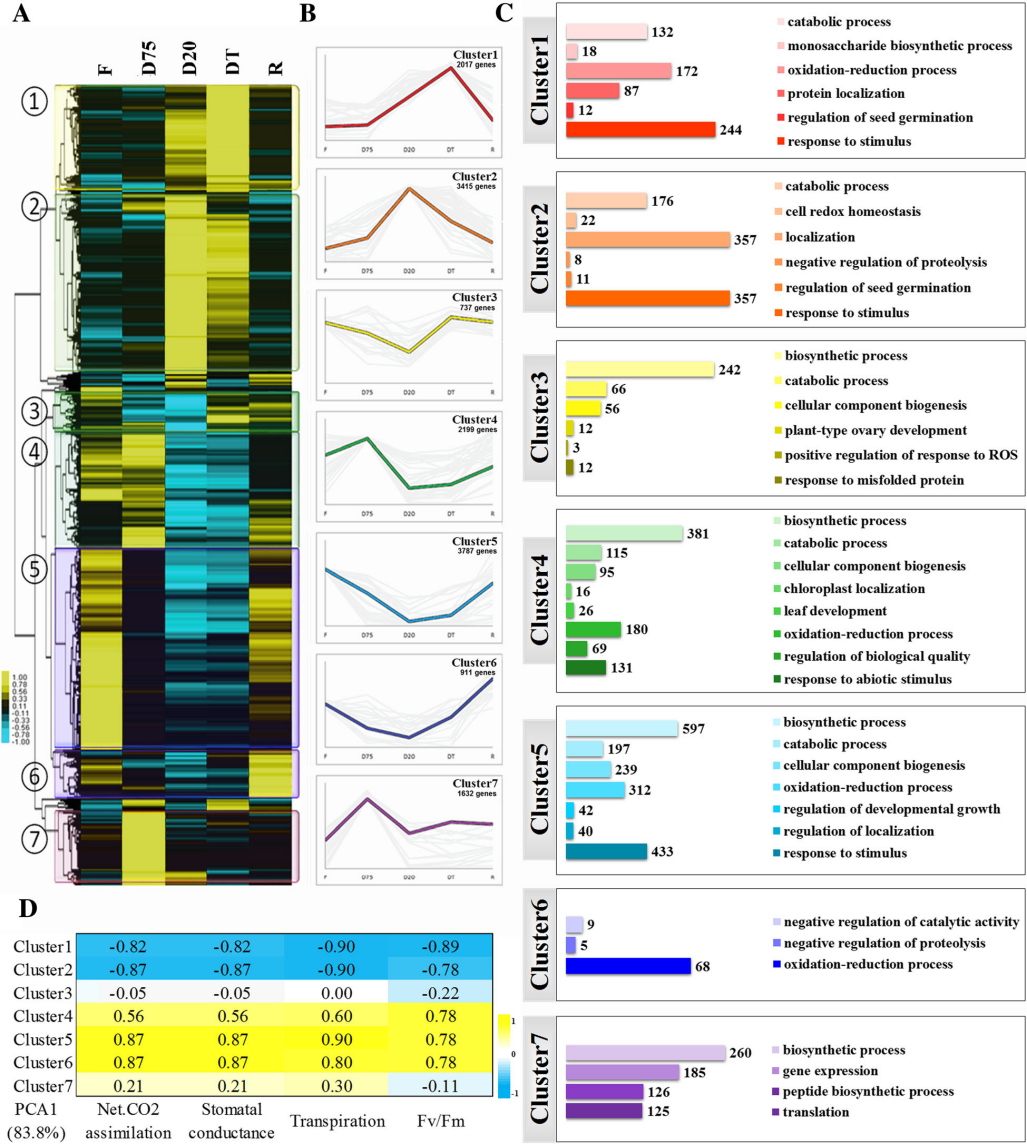
Expression profiles of DEGs and their correlations with physiological changes during dehydration. **a** Hierarchical clustering analysis using CLUSTER 3.0 to reveal the transcript expression patterns of DEGs in leaves subjected to different treatments (F, D75, D20, DT and R). Colored bars show the fold changes of gene expression in the dehydrated (D75, D20, DT) and rehydrated (R) leaves compared with fresh (F) leaves. **b** Representative curve of the seven clusters generated from hierarchical clustering analysis. **c** Biological processes were annotated to the seven clusters using Gene Ontology enrichment. **d** Correlation analysis of the seven clusters with physiological parameters using the first principle component data for each cluster. Sample abbreviations are the same as in Fig. 1

The hierarchical clustering of all DEGs in the five treatments showed significant differences in gene expression profiles. Therefore, we defined seven clusters of DEGs, along with a representative curve describing each transcript accumulation pattern (Fig. 3a, b). Transcripts peaking at the DT, D20 and D75 stages were represented as Clusters 1, 2 and 7, respectively, whereas transcripts repressed during dehydration (D75, D20 and DT) were found in Clusters 3, 4, 5 and 6. Interestingly, most dehydration-repressed transcripts reached their lowest levels at the D20 stage, regardless of whether they returned to control levels after rehydration (Cluster 5), or were transiently up-regulated at certain stages, such as D75 (Cluster 4), DT (Cluster 3) and R (Cluster 6).

The GO term enrichment of each cluster indicates that both metabolic processes (catabolism and biosynthesis) and developmental growth were repressed upon slight dehydration (Cluster 3), whereas responses to stimuli and redox processes/homeostasis were actively maintained until the water content decreased to 20% (Clusters 2, 4 and 5) (Fig. 3c). On the other hand, the transcripts activated at D75 (Cluster 7, translation and gene expression related) differed markedly from those activated at D20 and DT (Clusters 2 and 1, catabolic process, response to stimulus, redox process/homeostasis, localization, and regulation of seed germination).

To understand the significance of these gene expression clusters and to test the robustness of our cluster analysis, the first principle component from each stage was subjected to correlation analysis with four previously published physiological parameters [14]. We found that the decreases in net CO_2_ assimilation, stomatal conductance, transpiration and Fv/Fm during dehydration showed negative correlations with Clusters 1 and 2 and positive correlations with Clusters 5 and 6 (Fig. 3d).

### GO and KEGG enrichment of DEGs in dehydrated plants

**Fig. 4:**
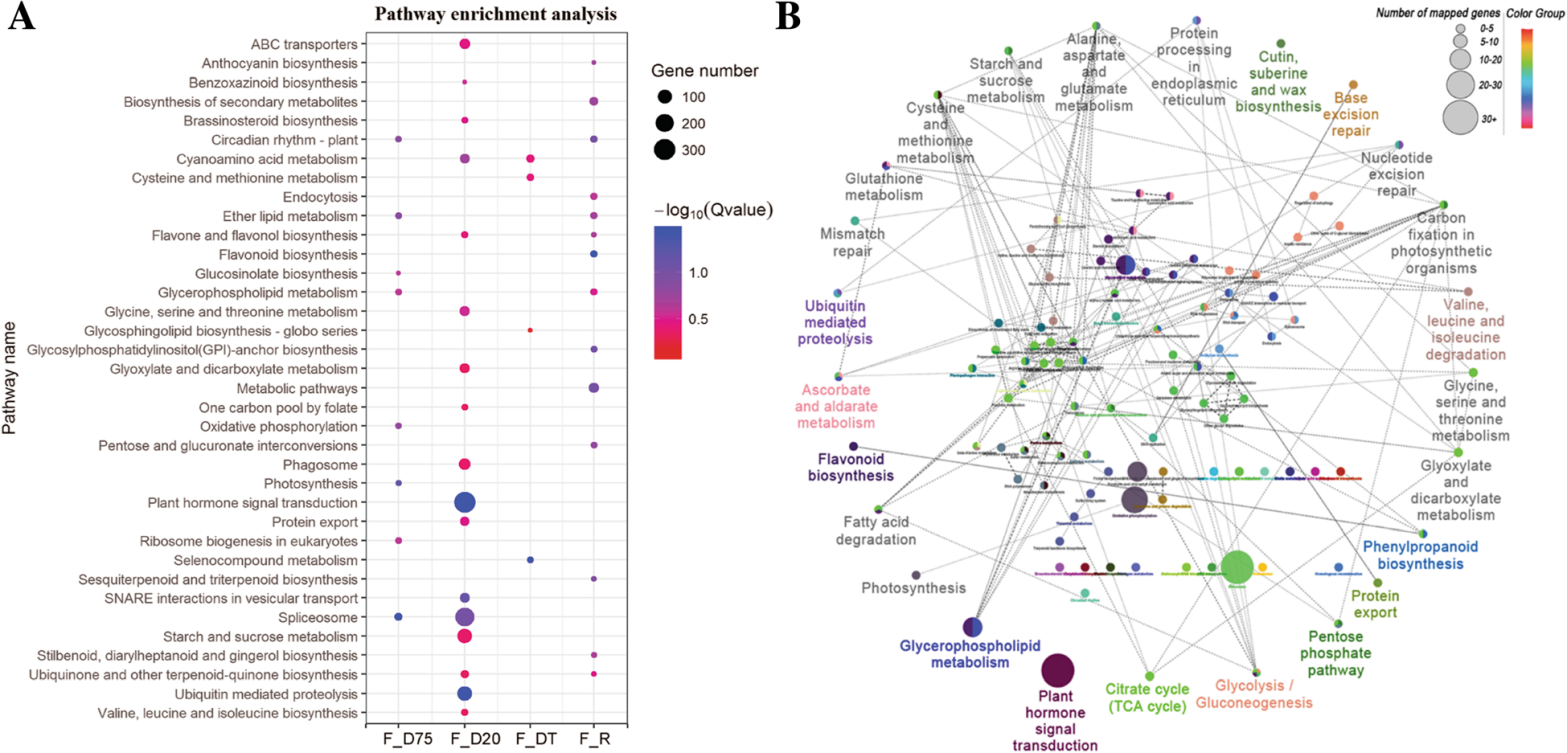
KEGG pathway enrichment and functionally organized GO/pathway term network of DEGs. **a** KEGG pathway analysis of the dehydrated (D75, D20, DT) and rehydrated (R) stages compared with the fresh stage (F). Significance is indicated by color, and the number of DEGs is indicated by the size of a circle. **b** Functionally organized GO/pathway term network against the KEGG pathway database using DEGs from the D20 stage. Sample abbreviations are the same as in Fig. 1

We performed KEGG analysis using the unigenes from each stage and observed the most significant enrichments in plant hormone signal transduction, spliceosome, starch and sucrose metabolism, and ubiquitin-mediated proteolysis pathways (Fig. 4a). Further GO and KEGG enrichment analyses were performed using the up- or down-regulated transcripts from each stage (Additional file 7: Table S5, Additional file 5: Fig. S2b). The GO terms electron transporter, chaperone binding, and iron-sulfur cluster binding were enriched among the transcripts induced by the D75, D20 and DT treatments, respectively, whereas developmental processes were enriched among the repressed transcripts during all dehydration stages (Additional file 7: Table S5). Metabolic pathways were also enriched among the transcripts that were repressed at all dehydration stages (Additional file 5: Fig. S2b). Interestingly, photosynthesis was enriched among not only the D20- and DT-repressed genes but also the D75-induced genes; plant hormone signal transduction was enriched among not only the transcripts that were decreased in all dehydrated samples but also in D20-induced transcripts. In addition, ubiquitin-mediated proteolysis, regulation of autophagy and protein processing in the endoplasmic reticulum (ER) were enriched among the transcripts induced by both D20 and DT. Notably, a large number of D20-specific DEGs associated with these pathways was not found in moderately dehydrated plants [5] (Additional file 2: Fig. S1d). This result prompted us to speculate that D20 (severe dehydration) is a crucial stage in transcriptional regulation of desiccation tolerance in *H. rhodopensis*.

Finally, we used these changes to construct a functionally organized GO/pathway term network based on the connectivity among GO terms (Fig. 4b, Additional file 8: Table S6). Numerous changes in this network were analyzed further, including primary and secondary metabolism, photosynthesis, hormone signal transduction, protein quality control and DNA repair.

### DEGs related to energy and primary and secondary metabolism

The expression patterns of photosynthesis-related genes generally were in agreement with the inhibition of photosynthesis observed during D20 and DT, including genes encoding the subunits of photosystem I (PSI) and photosystem II (PSII), light-harvesting complexes II and I, cytochrome b6/f complex and electron transport. Approximately a quarter of these transcripts, including NADPH-quinone oxidoreductase subunits and ATP synthase, were up-regulated only during the early stage of dehydration (D75). However, a few transcripts encoding ferredoxin 3 (FDX3) and subunits of the PSI and PSII reaction centers (psaA and psbC) maintained elevated expression levels throughout the dehydration process (Additional file 9: Fig. S3).

**Fig. 5:**
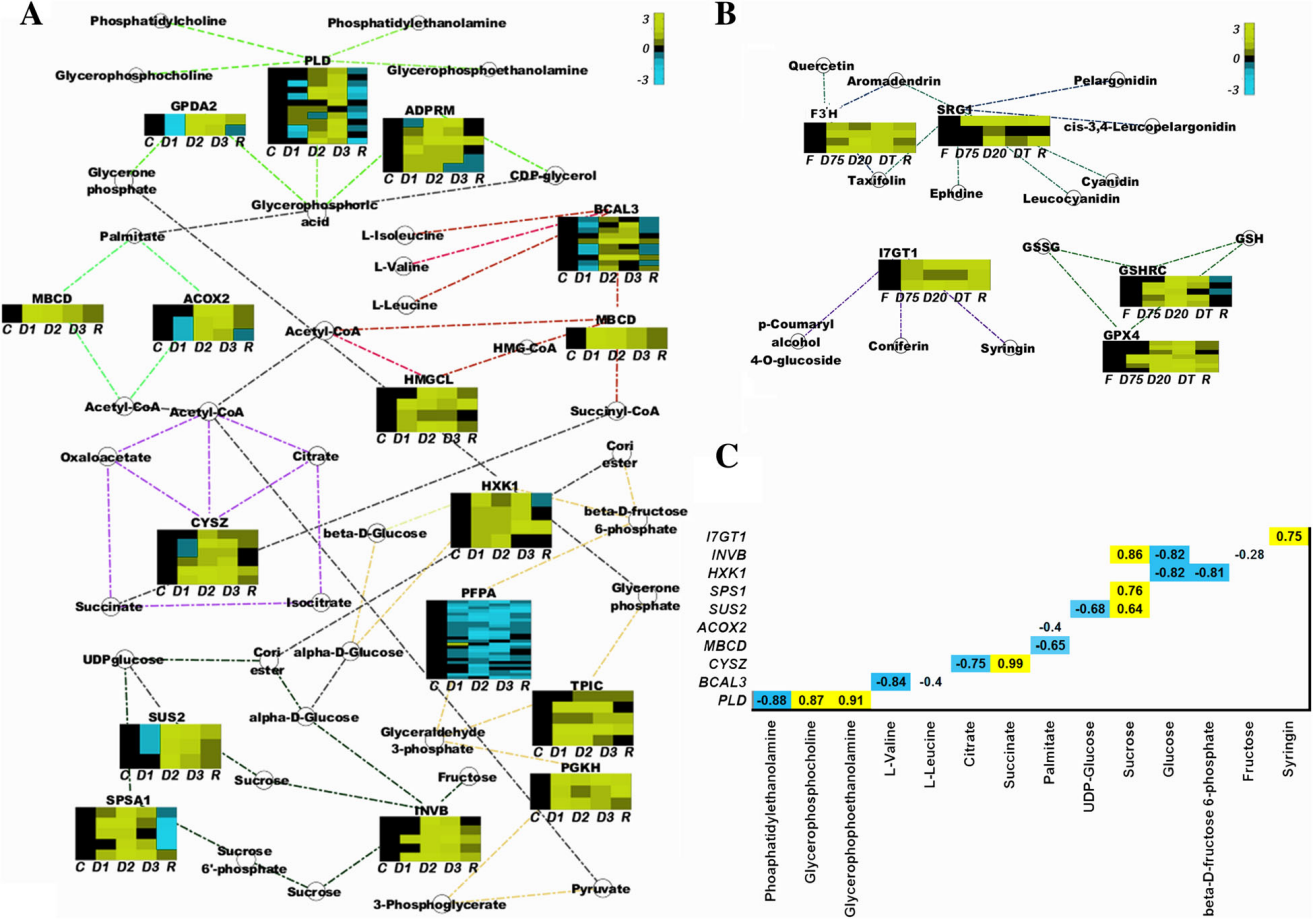
Expression heatmap of representative genes in the primary and secondary metabolism networks. Several KEGG pathways related to primary metabolism, including glycerophospholipid metabolism, starch and sucrose metabolism, valine, leucine and isoleucine degradation, fatty acid degradation, the TCA cycle and glycolysis (**a**), and to secondary metabolism, including phenylpropanoid biosynthesis, flavonoid biosynthesis and glutathione metabolism (**b**), are shown. Each associated gene is represented by a colored bar, showing the fold changes in the dehydrated (D75, D20, DT) and rehydrated (R) stages compared with fresh (F) plant material. **c** Correlation between transcript abundances and the corresponding metabolites during desiccation and recovery. The numbers indicate Pearson correlation coefficients, calculated using Cytoscape

The expression of genes related to metabolism was analyzed and closely compared with our previous metabolomics data [10–12]. A map was constructed of the D20-enriched KEGG pathways related to primary and secondary metabolism and the corresponding edges (significant co-expression relationship between two genes) (Additional file 10: Fig. S4). We found that the expression of phospholipase D (PLD), glycerol-3-phosphate dehydrogenase [NAD(+)], and manganese-dependent ADP-ribose/CDP-alcohol diphosphatase in the glycerophospholipid metabolic pathway was increased during desiccation. Two key enzymes involved in fatty acid degradation, 2-methylacyl-CoA dehydrogenase (MBCD) and acyl-CoA oxidase 2 (ACOX2), and enzymes involved in lipid degradation were also up-regulated. Branched-chain amino acid aminotransferase 3 (BCAL3) and hydroxymethylglutaryl-CoA lyase, involved in the valine, leucine and isoleucine degradation pathways, citrate synthase (CYSZ) involved in the citric acid (TCA) cycle, and enzymes involved in glycolysis/gluconeogenesis, such as triosephosphate isomerase (TPIC), phosphoglycerate kinase (PGKH) and hexokinase-1 (HXK1), were up-regulated, with the exception of the non-key enzyme pyrophosphate-fructose 6-phosphate 1-phosphotransferase subunit beta. Enzymes involved in sucrose synthesis, such as acid beta-fructofuranosidase and sucrose-phosphate synthase (SPSA1), were increased during dehydration, whereas sucrose synthase (SUS2) was elevated only in the D20 and DT treatments (Fig. 5a).

With respect to secondary metabolism, flavonoid 3′-monooxygenase (F3’H) and SRG1, which are involved in the accumulation of flavonoids, and flavanone 7-*O*-glucoside 2”-*O*-beta-L-rhamnosyltransferase (I7GT1), from the phenylpropanoid metabolism pathway, were up-regulated during dehydration. Two enzymes from the glutathione cycle, glutathione reductase (GSHRC) and phospholipid hydroperoxide glutathione peroxidase (GPX4), were also increased during desiccation (Fig. 5b).

Positive or negative correlations were found between the expression levels of most genes and the previously measured accumulation of their corresponding metabolites, depending on the reaction type (catabolic or anabolic) (Fig. 5c). For example, the levels of L-valine and leucine were negatively correlated with the expression of BCAL3, which plays a role in the catabolism of these amino acids, whereas SUS2 expression was correlated positively with sucrose content but negatively with UDP-glucose (Fig. 5c).

### DEGs involved in plant hormone signaling pathways

**Fig. 6:**
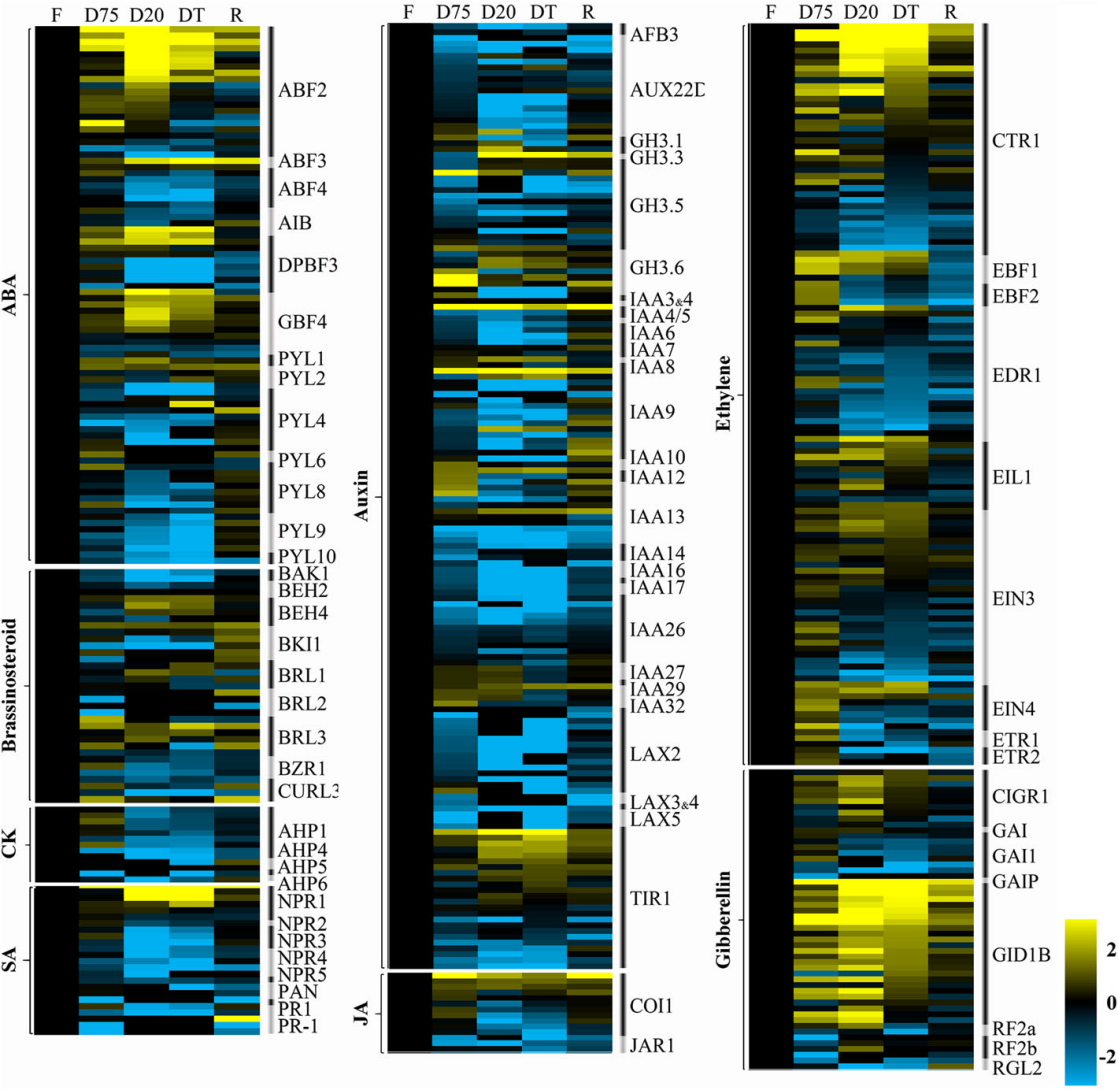
Heatmap representing transcriptional changes in the DEGs related to the plant hormone signaling pathway during dehydration (D75, D20, DT) and rehydration (R). Genes of interest were selected according to their relevance, and then the data were log transformed and normalized to those of the fresh stage to determine fold changes, as indicated by the colored bar

Transcripts encoding PYR/PYL/RCAR-type ABA receptors (PYL1 and PYL4), which are key factors (ABF2, ABF3, DPBF3/AREB3, GBF4 and AIB) in the ABA signaling pathway, and NPR1, a key factor in the SA signaling pathway, were highly induced during the D20 and DT stages (Fig. 6). Brassinosteroid (BR) receptors (BRL1 and BRL3) and BZR1 (BEH4), key factors in the BR signaling pathway, a JA receptor (COI1), an ethylene receptor (EIN4) and key regulators (CTR1, EDR1, EIN3 and EIL1/EIN3-like) in the ethylene signaling pathway, a gibberellic acid (GA) receptor (GID1B), an auxin receptor (TIR1), IAA-acyl acid-amido synthetases (GH3.3 and GH3.6), and negative regulators (IAA27, IAA9 and IAA13) in the auxin signaling pathway were all induced during dehydration. In contrast, positive regulators of the cytokinin signaling pathway AHP1 and AHP4 were up-regulated only slightly during the early stage of dehydration.

### DEGs related to protein quality control and DNA repair

**Fig. 7:**
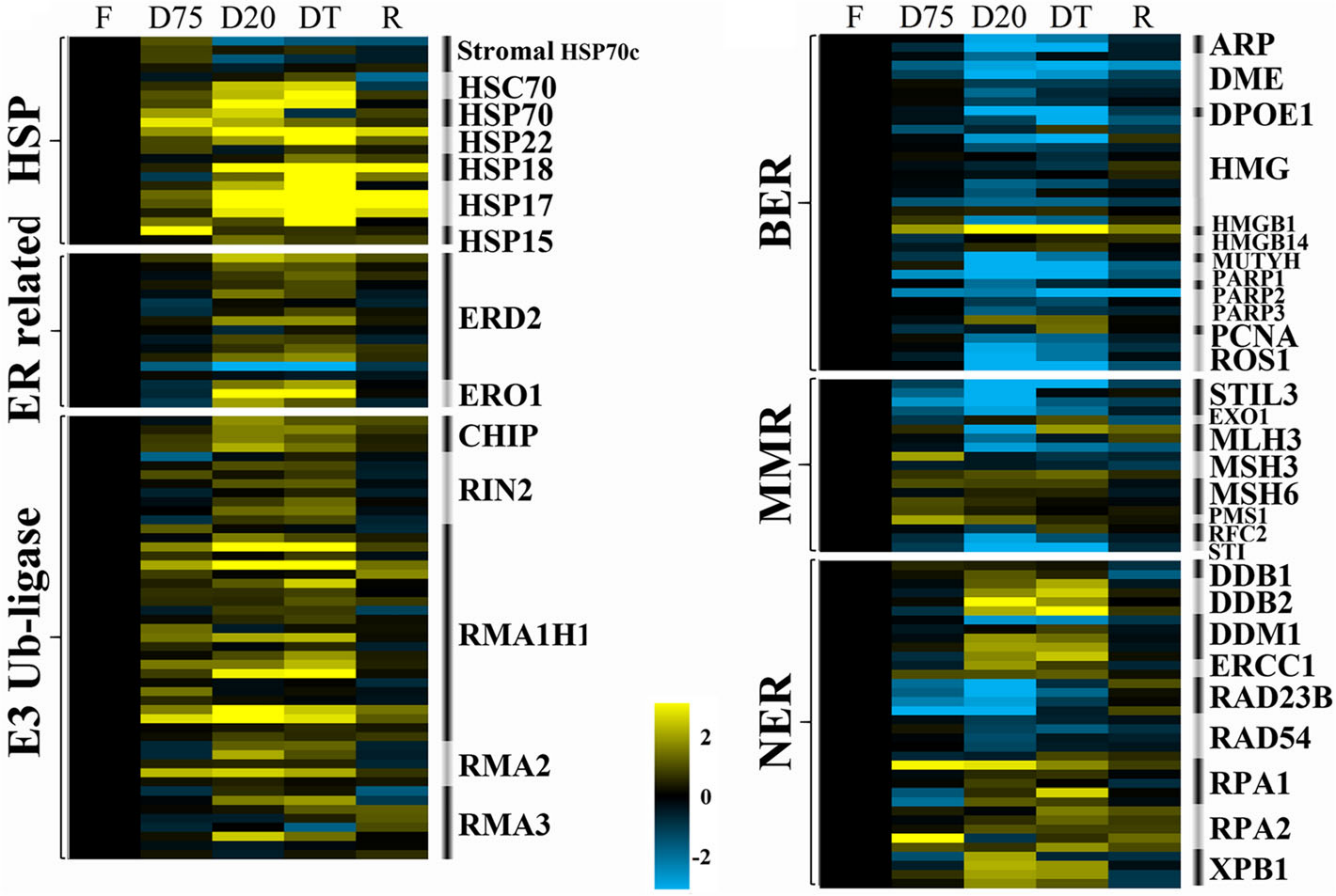
Heatmap representing transcriptional changes in the DEGs related to protein-quality control and DNA repair during dehydration (D75, D20, DT) and rehydration (R). **a** DEGs encoding heat shock proteins (HSPs), ER-related proteins and E3 ubiquitin ligases (E3 Ub-ligases). **b** DEGs involved in base excision repair (BER), nucleotide excision repair (NER) and mismatch repair (MMR). Data were log transformed and normalized to those of the fresh stage to determine fold changes, as indicated by the colored bar

Protein processing in the ER and ubiquitin-mediated proteolysis are both involved in protein-quality control. Among the related DEGs, most small heat shock proteins and HSP/HSC70s were most strongly up-regulated in the D20 treatment, whereas stromal HSP70c in chloroplasts was reduced in D20 and DT after transiently increasing in D75 (Fig. 7a). ER lumen protein-retaining receptor 2 (ERD2) and ER oxidoreductin-1 (ERO1) showed prominent up-regulation in the D20 and DT stages. The E3 ubiquitin-protein ligases, including the C terminus of HSC70-interacting protein, Rab interactor 2, RING membrane-anchor 1 homolog 1, and RING membrane-anchor 2 and 3, showed overall up-regulation from D75 onward, reaching the highest levels in D20 or DT.

Base excision repair (BER), nucleotide excision repair (NER) and mismatch repair (MMR) are the three main pathways involved in DNA repair. The majority of transcripts encoding proteins that participate in NER, including DNA damage binding proteins 1 and 2 (DDB1/DDB2), decrease in DNA methylation (DDM1), replication protein A 1 and 2 (RPA1/RPA2), and DNA repair helicase (XPB1) showed prominent patterns of up-regulation in D20 and DT (Fig. 7b). In contrast, most transcripts involved in BER and MMR were down-regulated during dehydration (Fig. 7b).

## Discussion

Most plants are not of the resurrecting type and therefore can tolerate water loss down to approximately 30% RWC only for relatively short periods [11, 15]. Below this water content, they are unable to recover even if water becomes available again; in other words, this level is the “point of no return” for most plants. Our previous physiological and metabolic studies described numerous changes that occurred in *H. rhodopensis* during severe dehydration [10, 12]. One of the aims of our present study was to understand whether and when (in terms of before or around this stage of dehydration) alterations occur at the transcriptome level in association with respective physiological changes.

It was previously shown that signaling and transcriptional changes induced by dehydration in *H. rhodopensis* regulate growth and photosynthesis (protein kinases and early light-inducible proteins), sugar metabolism (sucrose and raffinose synthase) and activation of diverse protectants (catalases, LEA and HSPs) [5]. Our de novo analysis not only supported these results but also identified novel genes that extend our understanding of the roles that cyclic electron flow (CEF), carbon turnover, phenylpropanoids, stress hormone signal transduction, protein quality control and DNA repair play in desiccation tolerance. The additional dehydration time points used here allowed us to outline the dynamic transcriptomic regulation during the dehydration and rehydration processes. The largest numbers of both up- and down-regulated DEGs were observed at this stage (Fig. 1). Most dehydration-repressed transcripts reached their lowest level at D20, regardless of whether these genes were transiently up-regulated during certain stages of dehydration or upon recovery (Fig. 3). A significant portion of the D20 up-regulated DEGs were annotated in the same biological processes and KEGG pathways as those up-regulated in DT, but did not always match those up-regulated in D75, as a large proportion of the DEGs showed opposing up- and down-regulation when the expression patterns of D75 and D20 were compared (Fig. 3). These data clearly indicate that desiccation tolerance mechanisms undergo reprogramming at the transcriptome level at the D20 stage. This finding extends the present understanding of desiccation tolerance mechanisms in *H. rhodopensis*.

### Energy and primary and secondary metabolic processes

Over-reduction of the electron transport chain and carbon starvation are among the main negative impacts of drought stress that result in reduced carbon fixation. Approximately 40% of the novel unigenes reported here are related to metabolic pathways and photosynthesis (Additional file 2: Fig. S1d). This large proportion allowed us to investigate the correlations of our transcriptomic data with physiological and metabolomic data (Fig. 3d; 5c). In this analysis, we identified changes in the photosynthetic apparatus and the mobilization of free and lipid-derived fatty acids and branched amino acids to ensure the availability of energy and alternative carbon sources to allow accumulation of osmoprotectants and antioxidants during desiccation. Many of these changes are similar to those that have been observed in plant seeds or during carbon starvation in non-resurrection plants [10, 16–22].

Resurrection plants can reduce linear electron transport flux through reorganization of the photosynthetic apparatus, thus preventing oxidative stress [10, 23]. A dehydration-triggered decrease in PSII performance was accompanied by reduced expression of associated genes, which belonged mostly to clusters 4, 5 and 6 (Fig. 3d), including chlorophyll a/b-binding protein and the light-harvesting complex Lhcb1, as reported previously [5]. In addition, the decrease in transcription of the PSI-K subunit of PSI (Additional file 9: Fig. S3) is directly responsible for the altered distribution of excitation energy and uncoupling between the photosystems [24], possibly explaining the observed decrease in electron flow between PSI and PSII [10, 25]. On the other hand, our present transcriptomic evidences indicating maintenance of CEF during dehydration corroborated the results of our previous study [10]. The up-regulation of ferredoxin and NADPH-quinone oxidoreductase subunits not only suggests the presence of the chloroplastic NAD(P)H dehydrogenase complex in *H. rhodopensis* but also highlights its role in desiccation tolerance as a component of CEF [26, 27].

The process of lipid turnover is very important in both tolerant and sensitive plants under drought stress. The up-regulated expression of PLD during dehydration (Fig. 5a) is likely to correspond with increased glycerophosphorylethanolamine (GPE) and glycerophosphorylcholine (GPC) and decreased phosphatidylethanolamine levels, reported in our previous study [10]. PLD activity induction by dehydration has been reported in the resurrection plant *Craterostigma plantagineum* [28], as well as in the drought-tolerant *Arabidopsis* ecotype Columbia [29]. A drastic decrease in lipid levels, especially membrane lipids such as phosphatidylethanolamine, has been observed in *Ramonda serbica*, a close relative of *H. rhodopensis* [25] and in the African resurrection plant *Sporobolus stapfianus* [26]. Thus, the reported correlation between PLD and related metabolites [10] (Fig. 5c), combined with the increased levels of malondialdehyde [5, 11, 30], strongly confirm the occurrence of lipid degradation in *H. rhodopensis* under stress. In comparison with the massive degradation of membranes in desiccation-sensitive species [31], these processes are quite limited in resurrection plants. Here, the turnover of damaged lipids may have a protective role, ensuring the accumulation of osmoprotectants such as GPC [32], signaling molecules and carbon precursors needed for biosynthetic processes during drought stress [10, 19, 33]*.* The up-regulation of the main enzymes responsible for fatty-acid degradation, MBCD and ACOX2 (Fig. 5a), supports the decreased free fatty acid content found during desiccation (Fig. 5c) [12].

Degradation of branched-chain amino acids provides an alternative carbon source in plants under unfavorable conditions [17, 21]. The up-regulation of BCAL3 and HMGL, which are involved in the degradation of valine, leucine and isoleucine (Fig. 5a), may explain the decreasing levels of these amino acids during dehydration (Fig. 5c) [10, 12].

Increased expression of *CYSZ*, *TPIC*, *PGKH*, *HXK1*, *SUS2* and *SPSA1* (Fig. 5a), which are related to the citric acid cycle, glycolysis/gluconeogenesis and sucrose biosynthesis, is in agreement with previous reports of increased metabolite levels during these biological processes and massive accumulation of sucrose during dehydration (Fig. 5c) [5, 10, 12].

We also found significantly elevated expression of genes involved in the synthesis of phenylpropanoids and flavonoids (Fig. 5b). I7GT1 encodes a UDP-glycosyltransferase, a type of enzyme that is related to the accumulation of p-coumaryl alcohol 4-*O*-glucoside, coniferin, syringin, and coniferyl and sinapyl alcohol 4-*O*-glucoside via glucosylation of soluble intermediates in *Arabidopsis* [34]. The up-regulation of F3’H and SRG1 during dehydration (Fig. 5b) is correlated directly with the accumulation of flavonoids, including quercetin, aromadendrin, taxifolin, ephedrine, leucocyanidin, cyanidin, *cis*-3,4-leucopelargonidin and pelargonidin [35, 36]. These phenylpropanoids and flavonoids are common plant secondary metabolites that function both as structural and signaling molecules [36].

Glutathione is involved in plant desiccation tolerance as an antioxidant [11, 37, 38]. DEGs related to glutathione metabolism have been reported previously in *H. rhodopensis* [5] and another resurrection plant species from the same family Gesneriaceae, *Boea hygrometrica* [3]. Over-expression of two transcripts in this study (Fig. 5b), GPX4 and GSHRC, accorded with these data and strongly confirmed the importance of glutathione as a major factor in desiccation tolerance.

### Plant hormone signal transduction

The plant hormone signaling network plays an integral role in the perception of and response to unfavorable environments. Previously, transient induction of JA and ABA has been reported, reaching their highest levels at approximately 55 and 25% RWC, respectively, in the dehydrated leaves of *H. rhodopensis,* whereas SA accumulates to its highest level at 25% RWC and is maintained during full dehydration [9]. The increased expression of biosynthesis- and signaling-related genes for these stress hormones contributes to plant drought tolerance by inducing biosynthesis of defense proteins and protective secondary metabolites. In this study, we found that the JA receptor COI1 was induced during dehydration (Fig. 6), but that the transcriptional activator of the JA signaling pathway, JAR1, was significantly repressed at the D20 and DT stages, indicating conversion of the JA signaling pathway under continuous stress, in accordance with the reported pattern of JA dynamics [9]. In contrast, at the transcriptional level, ABA and SA signaling transduction is activated at D20 and DT through the induction of key transcription factors such as ABFs, GBFs and AIB factors, and NPR1, thus confirming the roles of ABA and SA as stress signaling molecules and that of SA in the antioxidant response of the plant to dehydration [12].

Dynamic changes of ethylene in desiccation tolerance have not been reported; however, 1-aminocyclopropane-1-carboxylic acid oxidase transcripts have been shown to accumulate during dehydration [39]. In this study, the increased transcription of ethylene receptors (ETR1, ETR2 and EIN4) and the key transcription factor EIN3/EIL1 during the early stage of dehydration suggests a possible role of ethylene in the early response to dehydration, which is followed by repression of signal transduction in *H. rhodopensis,* as indicated by up-regulation of the key negative regulator CTR1 at the D20 and DT stages. Ethylene negatively regulates cell proliferation at the root apical meristem [40]. Thus, the transient increase of ethylene biosynthesis- and signaling-related genes in *H. rhodopensis* may contribute to the rapid inhibition of cell expansion during dehydration.

Crosstalk among SA, JA and ethylene-dependent signaling pathways regulates plant responses to abiotic and biotic stresses, which are frequently associated with hypersensitive response (HR)-like cell death [41–43]. The final response depends on the extent of crosstalk among these molecules. Inhibition of the ethylene and JA signaling pathways under severe and full dehydration and the crosstalk of these pathways with SA signal transduction likely contribute to prevention of HR-associated cell death, which is required for desiccation tolerance in resurrection plants.

Auxins, cytokinins, GAs and BRs have been recognized as crucial signaling molecules that control plant growth and development. The positive regulators of the cytokinin signaling pathway AHP1 and AHP4 were only slightly induced in the early stage of dehydration, whereas negative regulators of auxin pathways were induced throughout the dehydration process (Fig. 6), indicating inhibition of both signal transduction processes. This finding is in agreement with the observation of suspended growth of plants during dehydration. Notably, receptor genes for auxin, GA and BRs, including *TIR1*, *GID1B*, *BRL1* and *BRL3*, were up-regulated throughout the dehydration and rehydration processes, in parallel with IAA-acyl acid amido conjugators (group II GH3s) [44] and the BZR1 homolog BEH2, indicating a possible mechanism for the rapid recovery of the resurrection plant upon rehydration.

### Protein-quality control and DNA repair

The ability to maintain protein and DNA integrity is indispensable to cellular survival under constant exposure to adverse stresses that may result in mutagenesis or cell death [45]. Despite this importance, data on protein quality control and DNA repair are very limited or lacking, although recent molecular studies show their potential involvement in desiccation tolerance in the budding yeast *Saccharomyces cerevisiae*, *Arabidopsis* seeds and the resurrection plant *B. hygrometrica* [46–48]. The mechanisms controlling these processes, such as the unfolded protein response, aid in protein folding or in degradation of misfolded secretory proteins by producing protein-folding and other factors [49, 50]. Activation of the unfolded protein response in *H. rhodopensis* was reflected by up-regulation of two key regulators during D20 and DT (Fig. 7a): ERD2, a transmembrane ER lumen protein-retaining receptor that controls traffic in the Golgi body and retrograde transport to reclaim ER proteins [51], and ERO1, a protein associated with protein disulfide isomerase oxidoreductases, which play a key role in ER redox regulation via protein stabilization by forming disulfide bonds between structures [52].

The ubiquitin–proteasome system is the major pathway for proteasomal degradation of damaged and misfolded proteins. Activation of this system in D20 and DT, indicated by up-regulation of the majority of genes encoding E3 ubiquitin ligase (Fig. 7a), hypothetically results in proteomic redistribution; however, the reasons for this redistribution are unclear and remain to be explored in the future. A regulatory role of protein ubiquitination has been revealed, based on the accumulation of important metabolites during acquisition of rapid desiccation tolerance in *B. hygrometrica* [53]. E3 ubiquitin ligase may cooperatively interact with HSP/C70 for quality control [54, 55]. This interaction may occur intensively in *H. rhodopensis* during the D20 and DT stages.

Damaged DNA can be repaired via the processes of BER, MMR, and NER. In this study, we observed predominate activation of NER pathway genes, such as DDB1/DDB2, DDM1, RPA1/RPA2, XPB1 and PCNAs [56], indicating the importance of proofreading DNA repair to maintain genome integrity during severe dehydration. Studies of the DNA-damage response in seeds have suggested that particular proteins are required for certain repair mechanisms during the early stage of desiccation [47, 57]. Considering the similarity of desiccation tolerance in vegetative tissues of resurrection plants and seeds of non-recalcitrant species [58], a dynamic mechanism for maintaining genome integrity is therefore reasonably predicted in *H. rhodopensis* under desiccation. NER has been found to play an important role in reversing UV-induced DNA damage in plants [59]. Building upon known protective responses such as LEA and HSPs [5], this is the first report to associate NER with desiccation tolerance in resurrection plants. Further studies are needed to clarify the detailed mechanism of the DNA-repair process in *H. rhodopensis* under desiccation conditions.

## Conclusions

**Fig. 8:**
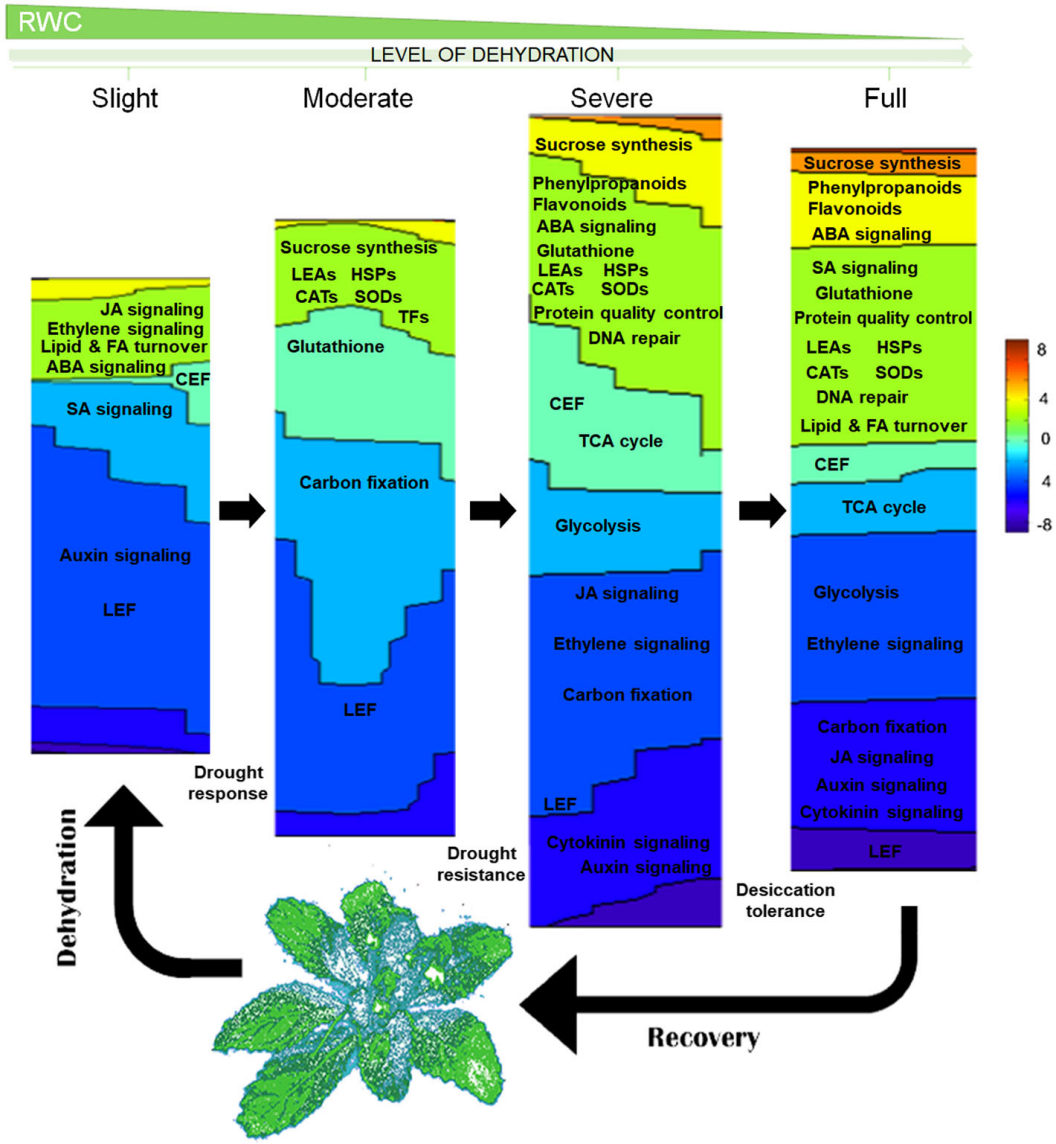
Conceptual model describing the transcriptional regulation of desiccation tolerance mechanisms in *H. rhodopensis* during dehydration. On the two-dimensional contour map, up- and down-regulated genes are represented as percentages for each stage. Fold changes are indicated by color. Representative pathways are labeled for each stage, including moderate dehydration, for which data from Gechev et al. (2013) [5] were used. JA, jasmonic acid; FA, fatty acid; ABA, abscisic acid; CEF, cyclic electron flow; SA, salicylic acid; LEF, linear electron transport flux; LEAs, late embryogenesis abundant proteins; HSPs, heat shock proteins; CATs, catalases; SODs, superoxide dismutases; TFs, transcription factors; TCA, citric acid cycle

The underlying difference between resurrection and non-resurrection plants under extreme drought remains somewhat unclear. We believe that our transcriptomic data contribute to the elucidation of new desiccation tolerance mechanisms in the Balkan endemic plant *H. rhodopensis*, as we have identified a large number of novel dehydration-responsive genes (Fig. 8). During the early stages of stress, the increased transcript levels of stress hormone signaling pathways initiate crosstalk among these signaling molecules, which is followed by fine tuning to avoid an increase in hypersensitive reaction-induced cell death as dehydration becomes severe. Consequently, further protective mechanisms related to energy, primary metabolism and secondary metabolism, protein quality control and DNA repair were transcriptionally mobilized during this stage. Activation of sucrose synthesis, lipid and fatty-acid turnover, HSP levels, LEA, NER, and the flavonoid, phenylpropanoid and glutathione pathways are among the main transcriptomic changes observed during desiccation. Notably, CEF and the mobilization of reserve carbon sources are maintained throughout the late stages of desiccation, in contrast to the linear decreases in electron transport and carbon uptake.

Together, our results show that during severe dehydration, a reprograming of the transcriptome occurs that defines the desiccation tolerance and resurrection behavior of *H. rhodopensis*.

## Materials and methods

### Plant materials and stress treatments

In vitro-propagated plants of *H. rhodopensis* [60] were transferred to a standard soil–sand–gravel mixture in pots (6 cm diameter) and grown for approximately 1 year in a controlled environment at 22–24 °C, under a 16-h photoperiod, 60% relative humidity, and a photon flux density of 36 μmol m^− 2^ s^− 1^.

Dehydration was induced by withholding water from potted plants. Plants required at least 8 days to become fully desiccated. After 10 days in the fully dry state, the plants were re-watered. Rehydration was initiated by watering the plants, and full recovery took 1 week. A non-destructive method of monitoring water content based on in vivo fast fluorescence was applied to collect plant leaf samples, according to the established relationship between leaf water content and photosynthetic performance during stress and recovery [10]. The Handy PEA (Hansatech Instrument Ltd., King’s Lynn, Norfolk, UK) device was used for measurement of the prompt chlorophyll *a* fluorescence of plants in the light-adapted state. Based on the SOM (self-organizing map) developed previously [10], we were able to collect samples at five time points corresponding to the following RWC stages: F (90% RWC), D75 (75% RWC), D20 (20% RWC), DT (6% RWC) and R (90% RWC). Mature, fully expanded leaves from the middle of rosettes of similar size and appearance were used in all measurements. At least four independent plants were used for each sampling point.

### RNA extraction, cDNA library construction, and Illumina high-throughput sequencing

Plant leaves (0.4 g) were ground under liquid nitrogen to a fine powder using a cooled mortar and pestle. Samples were placed in 2-mL Eppendorf tubes, and total RNA was isolated following the protocol for the GeneMATRIX Universal RNA kit (EURx Ltd., Gdansk, Poland).

Extracted RNA was used to construct cDNA libraries for sequencing analysis. Ten cDNA libraries were prepared using the Truseq™ RNA sample preparation kit from Illumina (San Diego, CA, USA). Libraries were size-selected for cDNA target fragments of 200–300 bp on 2% low-range ultra-agarose gels followed by PCR amplification using Phusion DNA polymerase (NEB, Ipswich, MA, USA) for 15 cycles. After quantification using the TBS380 fluorometer, 150-bp paired-end RNA-seq libraries were constructed using the Illumina HiSeq 4000 platform (Illumina).

### Transcriptome de novo assembly, sequence annotation, classification and alignment

The raw data were first processed using Seqprep (https://github.com/jstjohn/SeqPrep) and Sickle (https://github.com/najoshi/sickle) and the obtained high quality clean reads were subjected to de novo assembly using Trinity (https://github.com/trinityrnaseq/trinityrnaseq) [61]. The unique assembled transcripts were then used for functional annotation using BLASTX and BLAST2GO (http://www.blast2go.com/b2ghome) [62]. Metabolic pathway analysis was performed using KEGG (http://www.genome.jp/kegg) [63]. Sequence alignment was performed using BLAT [64] with contig sequences from both this de novo assembly and published data [5] (doi: 10.1007/s00018-012-1155-6, 18_2012_1155_MOESM1_ESM.xlsx). Unigenes were considered to differ when the sequence homology was < 90% in identity and < 80% in coverage.

### Differential expression analysis and **functional enrichment**

To identify DEGs between two different samples, the expression level of each transcript was calculated according to the FPKM method. RSEM (http://deweylab.biostat.wisc.edu/rsem/) [65] was used to quantify gene and isoform abundances. The R statistical software package Empirical analysis of Digital Gene Expression in R (http://www.bioconductor.org/packages/2.4/bioc/html/edgeR.html) [66] was utilized for differential gene expression analysis. Principle component analysis of DEGs was performed using SIMCA 13.0 (Umetrics, Satorius Stedim Biotech, Umeå, Sweden). In addition, functional enrichment analysis including GO and KEGG terms in comparison with the whole-transcriptome background was performed using a Bonferroni-corrected *p*-value < 0.05 threshold. GO functional enrichment and KEGG pathway enrichment were carried out using Goatools (https://github.com/tanghaibao/Goatools) and KOBAS (http://kobas.cbi.pku.edu.cn/download.php) [67]. For visualization of the functionally grouped network of KEGG terms, the ClueGO plugin [68] was used with Cytoscape [69]. To map all identified genes with altered expression in D20 and to visualize the pathway relationships among these genes, we used a general mapping method in which mapped genes represent a low percentage of all associated genes per pathway, and Bonferroni correction was omitted. Subsequently, the KEGG file for each pathway was downloaded, and all genes in the pathway were extracted from the ClueGO table. Then, network analysis was performed using Cytoscape software. The Pearson correlations between genes and metabolites were calculated using Cytoscape. The expression of each gene extracted from the ClueGO table for each pathway is expressed as the average log_2_ ratio for each treatment compared with the control.

### qPCR

All purified RNA samples described above were adjusted to the same concentration (100 ng/μL) using RNase-free ddH_2_O and then reverse-transcribed into cDNA using M-MLV reverse transcriptase (Promega, Madison, WI, USA), following the manufacturer’s instructions. Quantification of the transcripts was performed using SYBR® Green Realtime PCR Master Mix (TOYOBO, Osaka, Japan). Each reaction (10 μL) contained 0.4 μL of the cDNA template, 0.8 μL of the primer mixture (10 μM each, mixed to equivalent volume), 5.0 μL 2× PCR Master Mix and 3.8 μL ddH_2_O. Reactions were performed using a two-step method: 95 °C for 10 s, then 40 cycles of 95 °C for 10 s, 55 °C for 30 s, 72 °C for 30 s, and 95 °C for 15 s, followed by a final melt cycle from 55 °C to 95 °C. Gene-specific primers are shown in Additional file 6: Table S4. qPCR was performed for three technical replicates of each RNA sample (as well as two independent biological replicates). The normalized relative expression levels of the target genes were calculated by the 2^-ΔΔCt^ (ΔCt = Ct_Target_ – Ct_18S_, where Ct is the cycle threshold) using *18S* rRNA as the internal standard.

### Clustering analyses

The clustering of log-transformed expression data from each treatment was performed using Gene Cluster 3.0, with normalization of genes, the centered correlation similarity metric and complete linkage method. Clustered data were viewed, and the corresponding heatmaps were generated using JAVA TREEVIEW 1.6.4. Genes of interest were selected according to their relevance in particular biological processes or pathways, and then their expression data were pooled, log_2_ transformed and normalized to the untreated hydrated controls.

## Additional files

Additional file 1: **Table S1.** Summary of transcriptomic data of de novo assembly of fresh, dehydrated and rehydrated *Haberlea rhodopensis*. (XLSX 10 kb)

Additional file 2: **Figure S1.** Unigene analysis of *Haberlea rhodopensis* transcriptomic data*.* (a) Length distribution of all unigenes. (b) Species similarity distribution. (c) Distribution of the novel DEGs found in this de novo assembly that are unique or commonly shared to different dehydration time points. (d) Distribution of D20 unique genes in KEGG pathways. (TIF 859 kb)

Additional file 3: **Table S2.** Reads and base numbers of raw and clean sequences from ten libraries. (XLSX 10 kb)

Additional file 4: **Table S3.** Expression of genes that were known as dehydration inducible genes (ELIP, GS, beta-amylase, catalase, LEA etc.). (XLSX 22 kb)

Additional file 5: **Figure S2.** Confirmation of RNA-seq data using qRT-PCR and the KEGG analysis of up- and down-regulated transcripts. (a) Validation of RNA-seq data using qPCR. (b) KEGG analysis of up- and down-regulated unigenes of each stage. (TIF 721 kb)

Additional file 6: **Table S4.** Designed primers for the qPCR experiment in this article. (XLSX 11 kb)

Additional file 7: **Table S5.** GO enrichment of up- and down-regulated unigenes of each stage. (XLSX 13 kb)

Additional file 8: **Table S6.** Pathways identified from the functionally organized GO/pathway term network using DEGs in D20 stage. (XLSX 13 kb)

Additional file 9: **Figure S3.** Heatmap of expression patterns of transcripts encoding genes in photosynthesis. Data were log2 transformed and fold change is indicated by color bar. (TIF 1589 kb)

Additional file 10: **Figure S4.** Metabolic network of primary and secondary metabolism constructed by the ClueGO analysis. Mapped genes with changes in D20 are represented by color bar corresponding to the log2 fold change ratio between control and D20. The genes of interest linked to previously published metabolomics data are shown as array on the right. (TIF 734 kb)

## Abbreviations

ABA: Abscisic acid
DEGs: Differentially expressed genes
ER: Endoplasmic reticulum
GO: Gene Ontology
JA: Jasmonic acid
KEGG: Kyoto Encyclopedia of Genes and Genomes
NER: Nucleotide excision repair
PCA: Principal component analysis
