# Supplementary figures and images for: Transcriptome reprogramming during severe dehydration contributes to physiological and metabolic changes in the resurrection plant Haberlea rhodopensis
Source: BMC Plant Biol. 2018 Dec 13;18:351. doi: 10.1186/s12870-018-1566-0 (PMC6291977; doi:10.1186/s12870-018-1566-0)

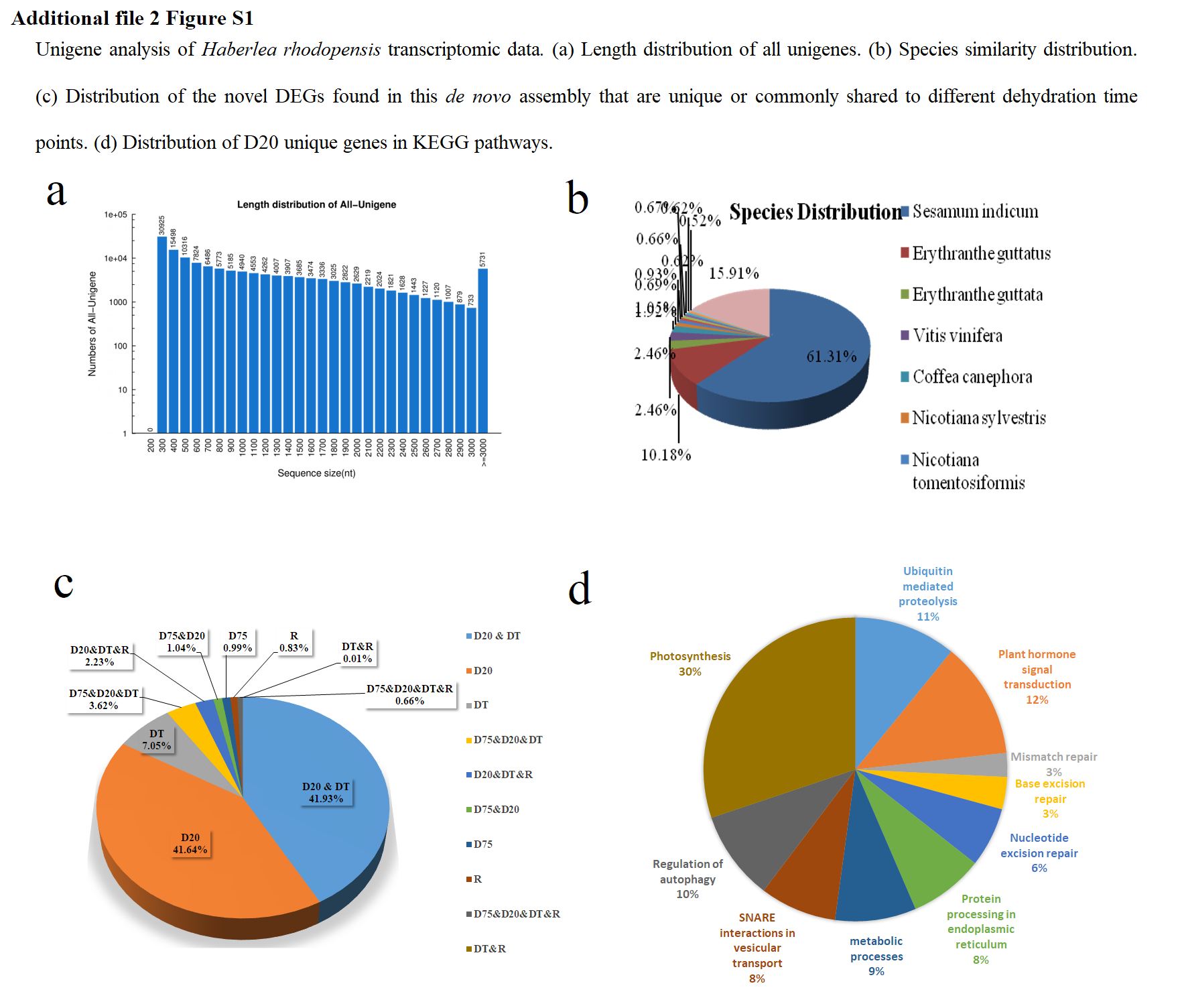

Supplement: Supplementary file 2 — Figure S1. Unigene analysis of Haberlea rhodopensis transcriptomic data. (a) Length distribution of all unigenes. (b) Species similarity distribution. (c) Distribution of the novel DEGs found in this de novo assembly that are unique or commonly shared to different dehydration time points. (d) Distribution of D20 unique genes in KEGG pathways. (TIF 859 kb) [file 12870_2018_1566_MOESM2_ESM.tif]

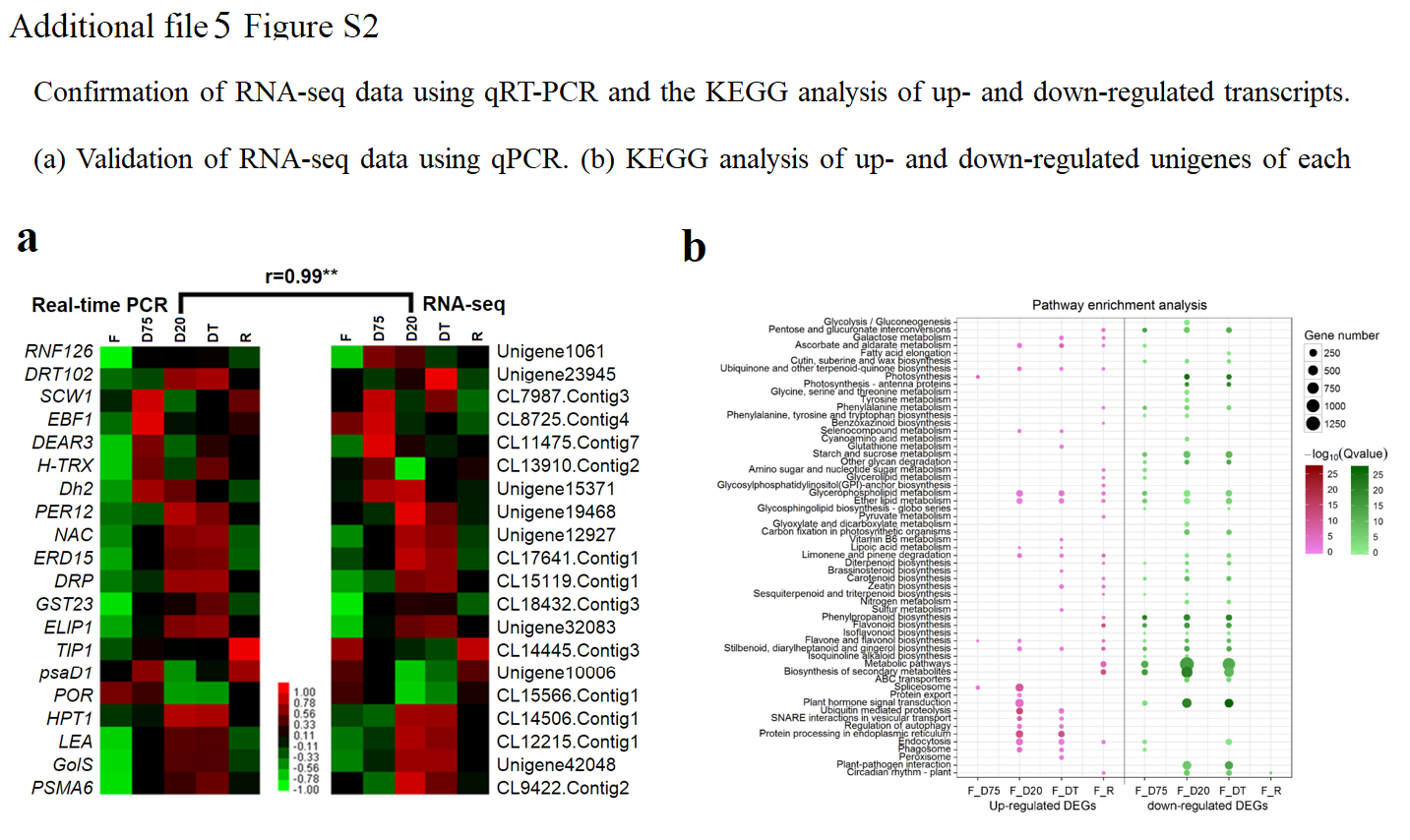

Supplement: Supplementary file 5 — Figure S2. Confirmation of RNA-seq data using qRT-PCR and the KEGG analysis of up- and down-regulated transcripts. (a) Validation of RNA-seq data using qPCR. (b) KEGG analysis of up- and down-regulated unigenes of each stage. (TIF 721 kb) [file 12870_2018_1566_MOESM5_ESM.tif]

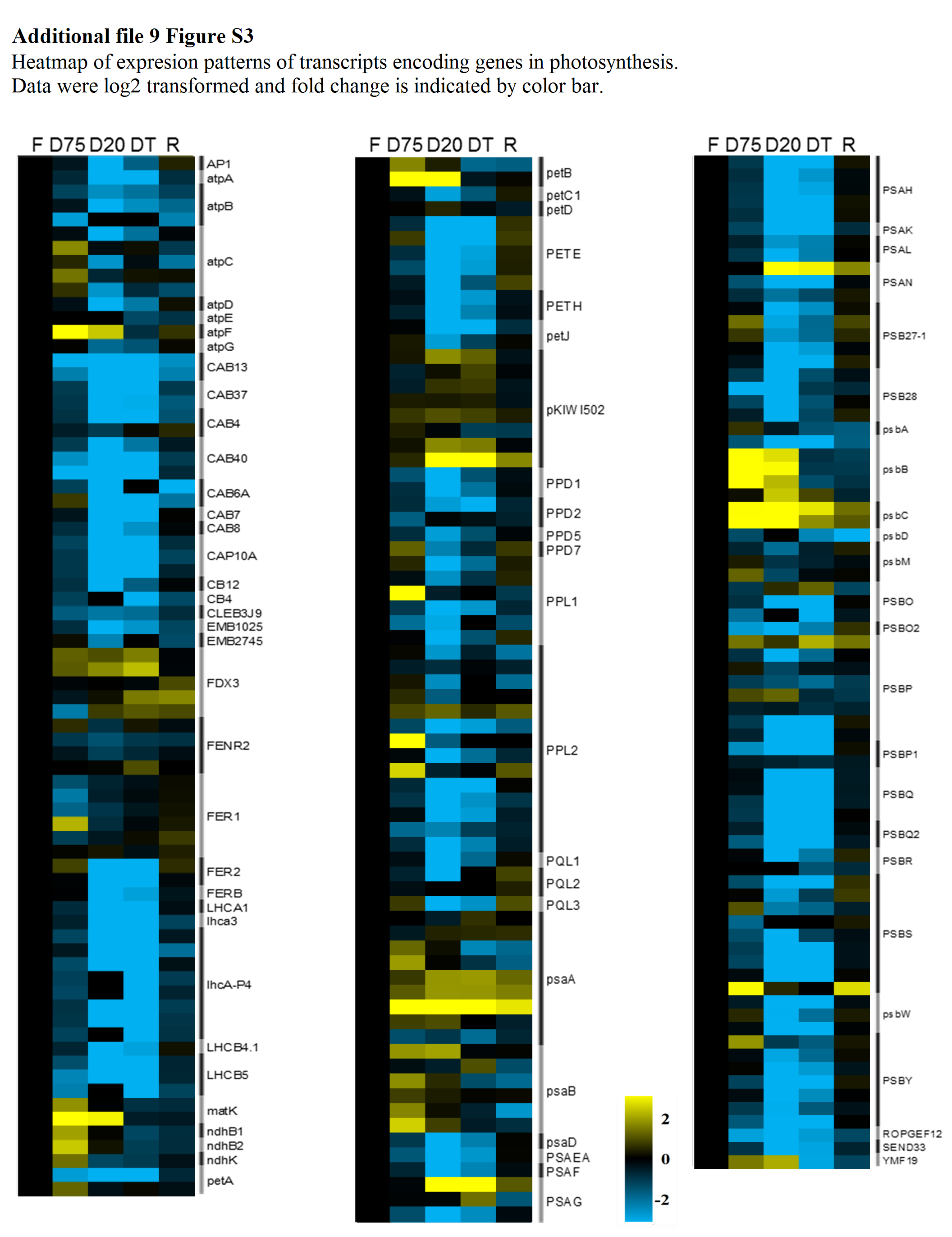

Supplement: Supplementary file 9 — Figure S3. Heatmap of expression patterns of transcripts encoding genes in photosynthesis. Data were log2 transformed and fold change is indicated by color bar. (TIF 1589 kb) [file 12870_2018_1566_MOESM9_ESM.tif]

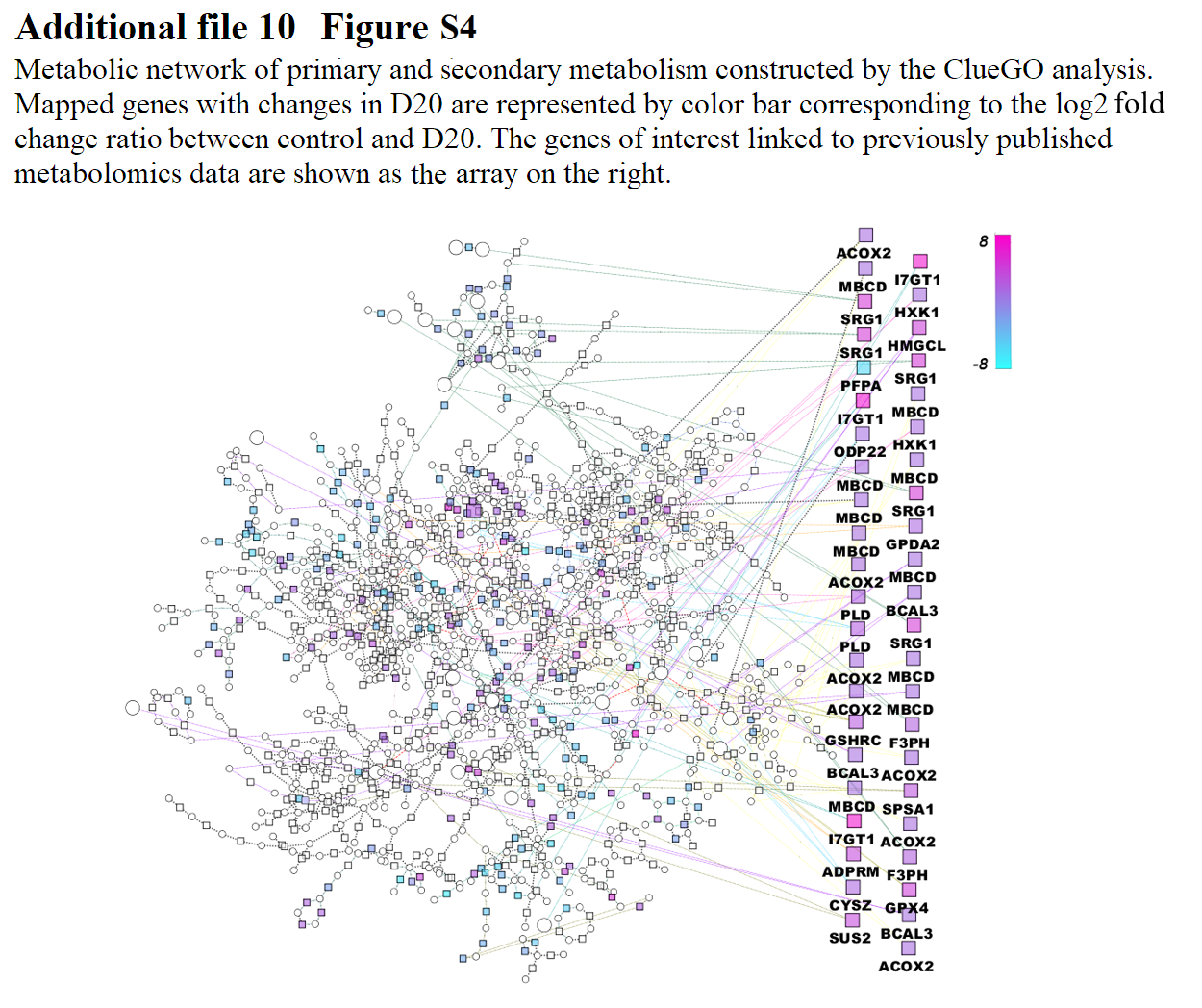

Supplement: Supplementary file 10 — Figure S4. Metabolic network of primary and secondary metabolism constructed by the ClueGO analysis. Mapped genes with changes in D20 are represented by color bar corresponding to the log2 fold change ratio between control and D20. The genes of interest linked to previously published metabolomics data are shown as array on the right. (TIF 734 kb) [file 12870_2018_1566_MOESM10_ESM.tif]
